# Analysis of genetic diversity among three *Triplophysa tenuis* populations by RAD-seq

**DOI:** 10.3389/fmolb.2024.1373754

**Published:** 2024-07-25

**Authors:** Wenqiong Wu, Junqiang Qiu, Yue Lin, Xike Li, Wenjuan Li, Yuanliang Duan, Yuanshuai Fu

**Affiliations:** ^1^ Key Laboratory of Freshwater Aquatic Genetic Resources, Ministry of Agriculture and Rural Affairs, Shanghai Ocean University, Shanghai, China; ^2^ Key Laboratory of Exploration and Utilization of Aquatic Genetic Resources, Ministry of Education, Shanghai Ocean University, Shanghai, China; ^3^ Fisheries Research Institute, Sichuan Academy of Agricultural Sciences, Chengdu, China

**Keywords:** *Triplophysa tenuis*, SSR, RAD-seq, genetic diversity, population structure

## Abstract

To investigate the genetic diversity of *Triplophysa tenuis* in the Shule River Basin of Gansu province, three populations were sequenced via RAD-seq technology. Twenty-nine microsatellite (SSR) markers with polymorphisms were finally screened to access the genetic diversity among the populations, of which 15 had high polymorphisms. The quantity of the alleles detected in the three populations of *T. tenuis* varied from 2 to 24. The locus with the most alleles was SSRC1, which had 24 alleles. Among the 29 SSRs, the range of effective allele number, observed heterozygosity, expected heterozygosity, and polymorphic information content were 1.246–16.615, 0.222–1, 0.198–0.940, and 0.178–0.937, respectively. Most of the identified loci were in the Hardy–Weinberg equilibrium. Analysis of the population structure revealed that the Yumen and Changma populations shared the same origin, while the Qiaowan population was different from them. The developed SSR markers discovered in this study will contribute to the conservation research on *T. tenuis* and the conservation of the fishery resources of the Shule River, providing scientific guidance for the development and utilization of *T. tenuis* resources and environmental protection.

## 1 Introduction

*Triplophysa* is widely distributed in the Qinghai–Tibet Plateau and its surrounding areas. It is a group of species that can adapt to the special climatic conditions of the plateau, such as low temperatures and hypoxia (Zhu, 1989; Wu and Wu, 1992). Scientists have confirmed up to 147 effective species of *Triplophysa* (He et al., 2011), including some synonymous species (Prokofiev, 2007), of which many have much unknown genetic diversity.

Genetic diversity is the cornerstone of species’ survival and evolution, and the higher the genetic diversity of a species, the stronger its adaptability to environmental changes (Liu, 2012). For most aquatic animals, rich genetic diversity is crucial to ensuring healthy population reproduction. This diversity enables individuals to better adapt to the environment and improve the survival competitiveness of the population when facing various pressures and threats. In addition, higher genetic diversity can offer a broader array of genetic resources and adaptation strategies, which are beneficial for species to survive and reproduce in complex and ever-changing environments. Therefore, studying, protecting, and maintaining the genetic diversity of aquatic species are of great significance for maintaining ecological balance and promoting sustainable development.

*Triplophysa tenuis*, studied here, belongs to the Cypriniformes order, Cobitidae subfamily, Nemacheilidae family, and *Triplophysa* genus. It has a strong adaptability to high altitudes and is naturally distributed in the Aksu River, Yerqiang River, and Tian River of the Tarim River Basin in southern Xinjiang (Zhu, 1989; Wu and Wu, 1992; Guo et al., 2012), as well as the Heihe and Shule rivers in Gansu Province (Zeng and Tang, 2010). *Triplophysa tenuis* is a dominant population in the distribution area and a significant component of fish diversity in the region, playing a crucial role in maintaining ecological stability. In recent years, due to the invasion of foreign species and human interference, endemic indigenous fish, such as *T. tenuis*, have become endangered (Ma et al., 2013). The reports on *T. tenuis* mainly focused on biology, growth characteristics, artificial reproduction, parasitic diseases, dietary analysis, mitochondrial DNA, etc. (Wang et al., 2015; 2016; Xie et al., 2018; Yao et al., 2018; Jin et al., 2020; Tian et al., 2022). At present, there are only two studies on the population genetics of *T. tenuis*, which focused on single-nucleotide polymorphism (SNP) development (Huo et al., 2022; Liu et al., 2022), and no research has been conducted on SSRs.

Since there are much unknown biodiversity in *Triplophysa*, we applied the RAD-seq technology to develop the microsatellite markers (SSRs) to detect the genetic diversity among three populations growing in the Shule River Basin. We aimed to offer scientific guidance for the development, utilization, and protection of *T. tenuis* wild resources and to effectively evaluate the germplasm status.

## 2 Materials and methods

### 2.1 Information on the sequenced samples

From 2019 to 2021, the individuals of *T. tenuis* from three distinct geographical locations in the Shule River Basin in Gansu Province were collected. A total of 44 fish were collected from Yumen (E96°49′07.23″; N40°15′57.06″); 26 fish were collected from Changma (E96°48′05.30″; N39°54′34.76″); and 36 fish were collected from Qiaowan (E96°43′15.19″; N40°33′59.37″). The muscle and fin tissues were collected and stored in 95% alcohol.

### 2.2 Extraction and preservation of DNA

The Ezup Column Animal Genomic DNA Purification Kit (Sangon Biotech (Shanghai) Co., Ltd.) was used to extract DNA of 106 *Triplophysa* samples, according to the instructions of the kit. The integrity of the extracted DNA was assessed via 1% agarose gel electrophoresis. The qualified DNA samples should have the light absorption ratio from 1.8 to 2.0 at A260/A280. Nine *Triplophysa* DNA samples were randomly selected from the Changma population, and three samples were mixed each to obtain a total of three samples (denoted as C1, C2, and C3). C1, C2, and C3 were used to develop SSRs through RAD-seq technology. Finally, the genetic diversity of the three populations (106 *Triplophysa*) was analyzed using the developed SSR markers.

### 2.3 Sequencing and quality control

The qualified DNA samples were successively treated by enzymatic digestion, random fragmentation, end repair, addition of A-tail, addition of sequencing adapters, purification, and PCR amplification to complete the library preparation, which were sequenced using the Illumina HiSeq PE1500 platform. The interfering information (such as sequencing adapter details, low-quality bases, and undetected bases) from the raw data was removed, resulting in the valid data.

### 2.4 Reference genome assembly and SNP detection

The sequencing data were clustered using CD-HIT-EST software (Li and Durbin, 2009). Based on the clustering results, each category was locally assembled using VelvetOpt software (Li et al., 2009) to obtain assembly sequences, which are contigs. Contigs below 100 bp were filtered to obtain the reference genome. Clean data were compared to the reference genome using BWA software (Li and Durbin, 2009), and duplicates were removed from the results using SAMtools (Li et al., 2009). Utilizing SAMtools for population SNP detection can help obtain high-quality SNP loci for developing SSRs.

### 2.5 Development of SSR markers

The RAD-seq result with the best sequencing quality of this study was selected for SSR development. SSRs were identified using SR Search software (Ruan et al., 2020). The search criteria are as follows: the repeat bases (two bases, three bases, four bases, five bases, and six bases) ≥ 5 times; the length of the PCR product was between 100 bp and 300 bp. The design of SSR primers was carried out using Primer Premier 5.0 software (Lalitha, 2000), with the following search criteria: 1) primer lengths between 24 bp and 36 bp; 2) Tm values of the primer between 46°C and 60°C; 3) Tm values of upstream and downstream were not exceeding 5°C in difference. Primers were synthesized by Novogene Bioinformatics Technology Co., Ltd.

### 2.6 Screening of SSRs by PCR-based sequencing

A total of 67 pairs of SSR primers were synthesized, and 20 genomic DNA samples of *T. tenuis* were randomly selected for PCR amplification to screen the optimal annealing temperature during the PCR reaction process. The PCR reaction system was 30 μL. The volumes of components are as follows: 14 µL of Premix Taq (2 × Taq PLUS MasterMix, CWBIO), 1 μL of DNA, 1 μL of the upstream primer (10 pmol/μL), 1 μL of the downstream primer (10 pmol/μL), and 13 µL of sterilized deionized water. The PCR reaction procedure is as follows: pre-denaturation at 94°C for 5 min; denaturation at 94°C for 45 s, annealing at a gradient annealing temperature (45°C–60°C) for 30 s, and extension at 72°C for 45 s and for 32 cycles; and extension at 72°C for 7 min.

After determining the optimal annealing temperature, 106 genomic DNAs of the individuals, respectively, collected from Qiaowan (36), Changma (26), and Yumen (44) were subjected to PCR amplification using these primers. The PCR products were detected via capillary electrophoresis (Beckman Coulter PA800 plus, Beckman Coulter, Inc.) to identify primers with polymorphisms. Except for the annealing temperature and DNA template, the PCR reaction system and procedure have not changed.

### 2.7 Genetic diversity analysis

Fluorescent primers were synthesized based on polymorphic loci by adding FAM at the 5’ end of the upstream primer. The newly synthesized primers were used to conduct PCR amplification on individuals of *T. tenuis* at three sampling locations, and the amplified DNA fragments were subsequently sequenced on the ABI 3730 sequencing platform. The sequencing data were used to analyze the genetic diversity of the three geographic populations located within the Shule River Basin.

### 2.8 Data analysis

GeneMarker^®^ (v1.95 Demo) software was used for preliminary processing and statistical analysis of typing data, which reveal a high polymorphism with the screened SSR primers. Genotyping was conducted by analyzing the length of gene fragments at each microsatellite locus. Cervus 3.0 software was applied to determine the allele number (*Na*), observed heterozygosity (*Ho*), expected heterozygosity (*He*), polymorphic information content (*PIC*), and Hardy–Weinberg equilibrium (Kalinowski et al., 2007). The Bayesian clustering analysis method of Structure 2.3.1 (Pritchard et al., 2000) was used to infer the population genetic structure, population genetic differentiation among different geographical distances, and the ancestry and structure of individuals in the population. The Bayesian ancestor identification method of Structure 2.3.1 was used to calculate the individual hybridization indexes and explore the mixing ratio of individuals in the population. Linkage disequilibrium analysis was conducted using Arlequin ver 3.5.2.2 (Excoffier et al., 2005).

The construction of the dataset was based on the Markov Chain Monte Carlo (MCMC) method, using an admixture model and an allele frequency correlated model. Structure software adopted a two-run mode: the first set *K* at 1–10; pre-burning period, Burnin = 100,000; iteration cycle, MCMC chain length = 100,000; each *K* was repeated 10 times to determine the source of alleles or genotypes of individuals in the population using the MCMC method at the maximum branch *K* value and perform ancestor identification. The Δ*K* method (Evanno et al., 2005) from the online Structure Harvester web v.0.6.94 (Earl and von Holdt, 2012) was used to estimate the optimal branch *K* values, which were corresponding to the maximum Δ*K* value is considered the best direct branch.

$$∆K=\frac{\text{Mean}\left(\left|L’’\left(K\right)\right|\right)}{\text{SD}\left(L\left(K\right)\right)},$$

where mean represents the average value and SD represents the standard deviation.

## 3 Results

### 3.1 Sequencing result analysis and SNP analysis

After sequencing and removing interference data, C1, C2, and C3 obtained 1,875,477,300 bp, 2,312,584,800 bp, and 2,460,561,600 bp raw data, and 1,875,477,300 bp, 2,299,109,100 bp, and 2,448,096,300 bp clean data, respectively. The effective rates (clean data/raw data) of C1, C2, and C3 reached 99.35%, 99.42%, and 99.49%, respectively. However, Q20 and Q30 of C1 reached 97.86% and 93.50%, respectively, which were higher than those of C2 (97.78% and 93.23%) and C3 (96.95% and 91.38%), indicating that C1 had the highest sequencing quality (the raw data of C1 was stored in the GenBank Short Read Archive database, accession number: PRJNA1083026). Moreover, the GC contents of C1, C2, and C3 were 40.22%, 39.19%, and 38.78%, respectively.

**FIGURE 1:**
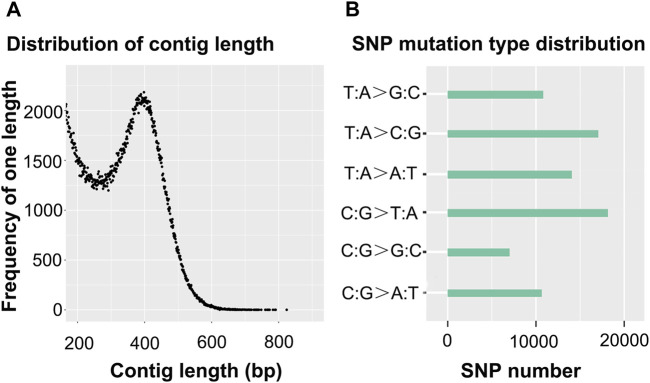
Distribution of the contig length **(A)** and SNP mutation-type distribution **(B)**.

In this study, the total length of reference genes was 187,821,632 bp, which was obtained through clustering and RAD partial assembly, and, the GC content was 38.31%. The total contig number was 580,217. The distribution of contig length is shown in Figure 1A, and its average contig length and N50 length were 323 and 375, respectively.

In C1, the total number of reads of valid sequencing data was 12,421,988, and the number of reads mapped to the reference genome was 10,970,937, resulting in an 88.32% mapping rate. The percentage of the reference genome with at least one base covering the site was 36.63%. Furthermore, 26.59% of the reference genome has at least four base coverage sites.

Sequencing obtained 77,806 SNP loci, of which 35,238 loci were derived from transitions and 42,568 loci were derived from transversions, and the ratio of heterozygous SNP loci to the total number of SNP loci was 0.274%. In this study, six types of mutations appeared, which were T-mutated into G and A-mutated into C (denoted T:A > G:C mutation type), T-mutated into C and A-mutated into G (denoted T:A > C:G mutation type), T-mutated into A and A-mutated into T (denoted T:A > A:T mutation type), C-mutated into T and G-mutated into A (denoted C:G > T:A mutation type), C-mutated into G and G-mutated into C (denoted C:G > G:C mutation type), and C-mutated into A and G-mutated into T (denoted C:G > T:A mutation type). Only the number of the C:G > G:C mutation type was less than 10,000, and the number of the C:G > T:A mutation type and the T:A > C:G mutation type was more than 15,000. The largest number of SNP mutation types was C:G > T:A. More detailed information is shown in Figure 1B.

Note: A) The abscissa represents the contig length. The ordinate represents the frequency of one length. B) The ordinate represents the mutation type. With T:A > C:G as an example, this type of SNP mutation includes T mutated into C and A mutated into G. Due to the fact that sequencing data can be compared to both the positive and negative strands of the reference genome, when T mutated into C-type mutations appeared on the positive strand of the reference genome, A mutated into G-type mutations were located at the same position as the negative strand of the reference genome. Therefore, T mutated into C and A mutated into G are classified into one group.

### 3.2 SSR analysis of polymorphisms

**FIGURE 2:**
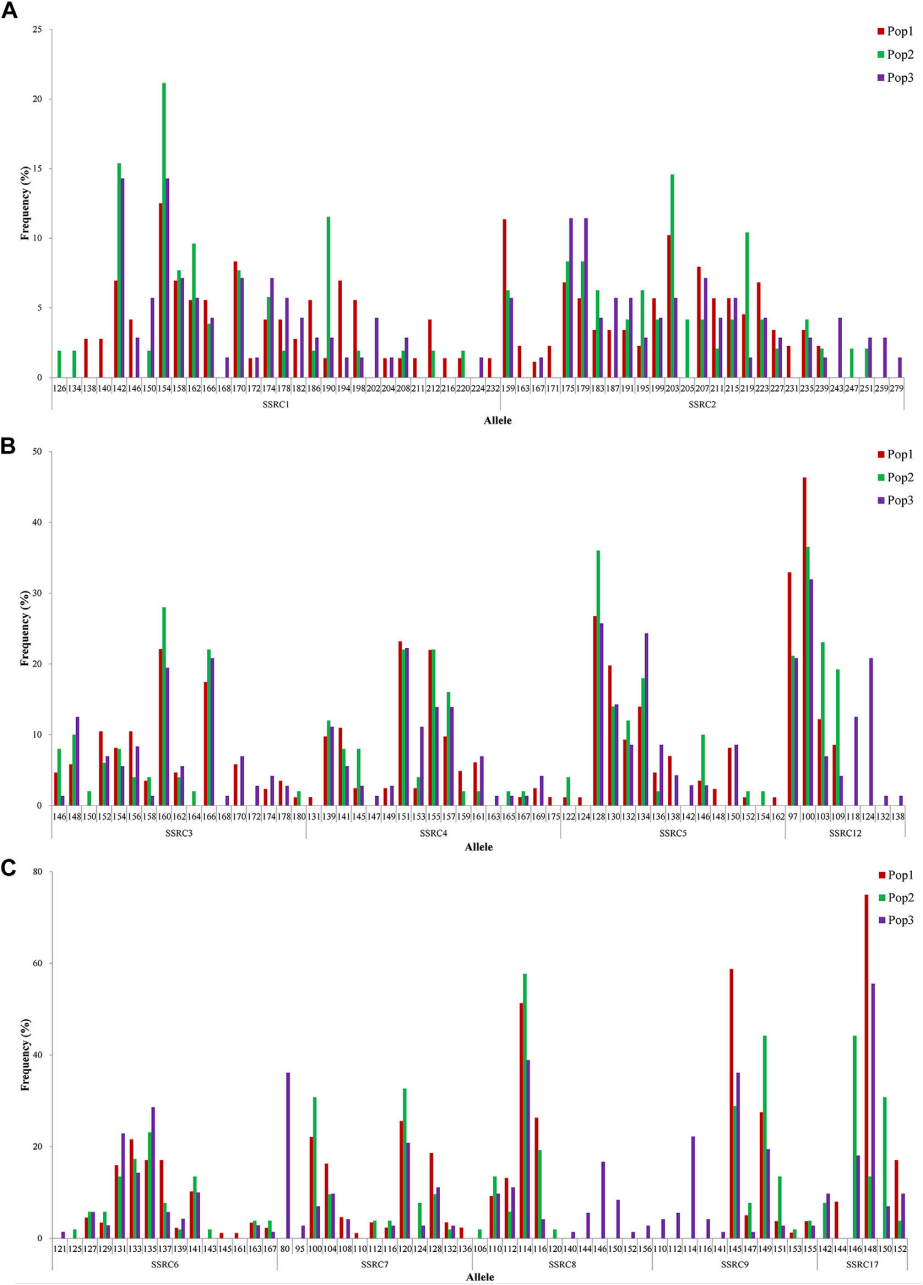
Allele frequency of SSRs in *Triplophysa tenuis*. Note: Pop1, Pop2, and Pop3 represent the population in Yumen, Changma, and Qiaowan, respectively. Here, 142 represents the appearance of SSRC1 at base 142 of the sequence; the same applies to others. The ordinate represents the appearance frequency of SSRs.

According to the specific SNPs, a total of 67 SSR loci were obtained, and 29 SSRs with polymorphisms were obtained by further screening, which were SSRC1–SSRC29, and more detailed information is presented in Supplementary Table S1. The distribution of polymorphisms for each SSR in three populations is illustrated in Figure 2 and Supplementary Figure S1. The number of SSR loci in the three populations ranged from 3 to 30. Among them, there were 10 SSRs with more than 10 mutation loci, which were SSRC1–SSRC10, especially when the number of SSRC1 and SSRC—2 mutation loci—reached 30 and 27, respectively. Most of the potential mutation loci were present in the three populations (SSRC1 in 142 from Figure 2A; SSRC3 in 146 from Figure 2B; and SSRC6 in 127 from Figure 2C), albeit with varying probabilities. Very few variation loci were unique to a population, such as SSRC1 only appeared in the locus of 138 from the Yumen population (Figure 2A), SSRC3 only appeared in the locus of 150 from the Changma population (Figure 2B), and SSRC6 only appeared in the locus of 121 from the Qiaowan population (Figure 2C). In addition, 29 SSRs had a maximum of 45 unique mutation loci in the Qiaowan population, while the Yumen and Changma populations had only 21 and 23 unique mutation loci, respectively.

### 3.3 Genetic correlation analysis of polymorphic SSRs in three populations

The genetic diversity results of *T. tenuis* in the three geographic populations are shown in Table 1 and Supplementary Table S2. The greatest diversity was observed in Qiaowan population, which had the average *Ne*, *Na*, *Ho*, *He*, and *PIC* values at 4.213, 7.379, 0.657, 0.651, and 0.605, respectively, compared to 3.998, 6.483, 0.652, 0.598, and 0.549, respectively, in Yumen and 3.691, 6.414, 0.645, 0.638, and 0.574, respectively, in Changma. The number of alleles in the three populations ranged from 2 to 24. Specifically, the allele count was 2–24 in the Yumen population, 3–19 in the Changma population, and 2–22 in the Qiaowan population. In addition, the locus with the most alleles was SSRC1, which had 24 alleles in the Yumen population. Among the three populations, SSRs with alleles above 10 were SSRC2, SSRC3, SSRC6, SSRC4, and SSRC1. The *Ne* values in the three populations ranged from 1.246 to 16.615. The largest number of *Ne* in the Yumen population was SSRC1 with 16.615, while in the Changma and Qiaowan populations, they were SSRC2 with 13.880 and SSRC2 with 16.225, respectively. The *Ho* values of Yumen, Changma, and Qiaowan populations ranged from 0.222 to 1, 0.320 to 1, and 0.286 to 1, respectively. In the Yumen, Changma, and Qiaowan populations, the *He* values ranged from 0.198 to 0.940, 0.341 to 0.980, and 0.322 to 0.938, respectively. The *PIC* values in the Yumen, Changma, and Qiaowan populations ranged from 0.178 to 0.937, 0.295 to 0.923, and 0.275 to 0.935, respectively. In the population of Yumen, there were 2 loci with *PIC* values less than 0.25, 9 loci with *PIC* values between 0.25 and 0.5, and 18 (62.07%) loci with *PIC* values greater than 0.5. In the population of Changma, there were 10 loci with *PIC* values between 0.25 and 0.5 and 19 (65.52%) loci with *PIC* greater than 0.5. In the population of Qiaowan, there were 9 loci with *PIC* values between 0.25 and 0.5 and 20 (68.97%) loci with *PIC* values greater than 0.5. For the *PIC* analysis in the three populations, 15 out of the 29 loci (SSRC1–SSRC3, SSRC5–SSRC10, SSRC12–SSRC15, SSRC23, and SSRC24) had values greater than 0.5, while 6 loci had values less than 0.5. Furthermore, the most extreme values for all indicators were observed in the Yumen population. For the Hardy–Weinberg equilibrium testing, in Yumen population, there were five SSR loci with significant *p*-values; in Changma population, there were eight SSR loci with significant *p*-values; and in Qiaowan population, there were six SSR loci with significant *p*-values. SSRC11, SSRC17, SSRC19, and SSRC29 with significant *p*-values were found in at least two populations. Noticeably, SSRC2, SSRC7, SSRC22, and SSRC23 were in a non-linked state in the Yumen population, and SSRC17, SSRC19, SSRC22, SSRC23, and SSRC28 were in a non-linked state in the Qiaowan population, and only SSRC10 was in a non-linked state in the Changma population (Supplementary Tables S3–S5).

**TABLE 1:** Genetic diversity of the three populations.

Pop	Locus	*N*	*Ne*	*Na*	*Ho*	*He*	*PIC*	*P*_*HWE*_
Pop1 (Yumen)	SSRC3	43	8.2	13	0.814	0.878	0.867	**
	SSRC6	44	6.841	12	0.864	0.854	0.837	**
	SSRC14	44	3.108	7	0.614	0.678	0.622	*
	SSRC19	44	2.004	3	0.455	0.501	0.444	*
	SSRC29	36	2	2	1	0.5	0.375	**
Pop2 (Changma)	SSRC5	25	4.789	9	0.88	0.791	0.766	**
	SSRC11	26	2	3	0.462	0.5	0.408	**
	SSRC16	26	1.899	6	0.462	0.473	0.428	**
	SSRC17	26	3.166	5	0.692	0.684	0.632	*
	SSRC18	26	2.715	5	0.423	0.632	0.583	*
	SSRC19	25	2.252	4	0.44	0.556	0.508	**
	SSRC24	26	2.704	4	0.346	0.63	0.565	**
	SSRC29	25	2.08	3	1	0.519	0.404	**
Pop3 (Qiaowan)	SSRC8	36	4.679	10	0.639	0.786	0.765	**
	SSRC9	36	4.423	10	0.528	0.774	0.744	*
	SSRC11	36	4.019	8	0.444	0.751	0.722	**
	SSRC12	36	4.73	8	0.472	0.789	0.758	**
	SSRC17	36	2.74	5	0.472	0.635	0.598	**
	SSRC29	36	1.976	2	0.889	0.494	0.372	**

Note: N represents the total number of samples. *Ne* represents the number of effective alleles. *Na* represents the number of alleles. *Ho* represents the observed heterozygosity. *He* represents the expected heterozygosity. *PIC* represents the polymorphism information content. *P*_*HWE*_ represents the Hardy–Weinberg equilibrium test (with Bonferroni correction). Significant values (* or **) indicate deviance from HW expected proportions at these values.

**FIGURE 3:**
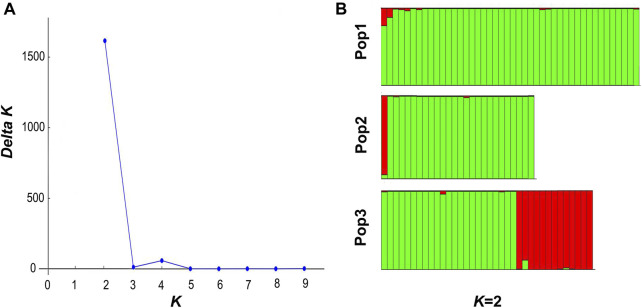
Genetic structure analysis of *T. tenuis* populations. Note: **(A)** *K*-value structure analysis based on the In *P* (D) model. *K* represents the number of possible population branches of the sample. The Δ*K* method was used to estimate the optimal branch *K* values, which were corresponding to the maximum Δ*K* and is considered the best direct branch. **(B)** Different colors represent different genetic compositions. The ordinate represents the genetic proportion (0%–100%) of each individual. Pop1, Pop2, and Pop3 represent the population in Yumen, Changma, and Qiaowan, respectively.

Based on the MCMC method, the analysis of the optimal group classification values exhibited two groups in the optimal state when *K* was at 2 (Figure 3A), which suggested that there was no significant geographical clustering among the populations. The resulting data in the genetic composition source analysis revealed that the Yumen and Changma populations probably shared the origins, while the Qiaowan population was different from them. A certain degree of genetic differentiation among the three geographical populations could be inferred (Figure 3B).

## 4 Discussion

As a member of numerous *Triplophysa* species, *T. tenuis* plays an important role in the genetic diversity of fish. The research on genetic information of fish is of great significance in fish population distribution, resource conservation, and other aspects. RAD-seq technology is widely used in identifying high-quality mutation loci in animals and plants (Bus et al., 2012; Chen et al., 2020). In this study, 29 SSRs with polymorphisms were developed using RAD-seq technology combined with SSR development technology to analyze the genetic diversity of *T. tenuis*. This provides a foundational dataset for the conservation of *T. tenuis* fishery resources.

In the process of analyzing biological genetic diversity, *PIC* is an important indicator. When the *PIC* value is less than 0.25, it is considered a low-degree polymorphic locus; when 0.25 < *PIC* < 0.5, it can be classified as a moderately polymorphic locus; and when the *PIC* value is greater than 0.5, it is considered a highly polymorphic locus (Botstein et al., 1980). Huo et al. (2022) utilized SNPs to analyze the *PIC* value, which averaged only 0.1139, of *T. tenuis* in the Tarim River Basin in Xinjiang province. They discovered that multiple populations in the basin exhibited high genetic diversity. In this study, the *PIC* values of the three *T. tenuis* populations were all higher than 0.5, ranging from 0.549 to 0.605. This indicates that the *T. tenuis* populations from the Shule River Basin in Gansu province exhibit high genetic diversity, which is greater than that of *T. tenuis* in the Tarim River Basin in Xinjiang province. In addition, species with richer genetic diversity have higher heterozygosity and stronger adaptability to the environment (Qin et al., 2013). In this study, the observed and expected heterozygosity of the Qiaowan population was higher than that of the Yumen and Changma populations, indicating that the Qiaowan population may have higher environmental adaptability.

Previous studies have shown that environmental differences, geographic distance, and other factors can lead to genetic isolation in populations, reducing the likelihood of successful migration (Hamrick and Godt, 1996; Chen and Wang, 2021; Gai et al., 2021). Huo et al. (2022) analyzed the population clustering phenomenon of *T. tenuis* in various regions of the Tarim River Basin in Xinjiang province using sampling distance. In this study, the sampling points of the Yumen and Changma populations were connected through artificial channels, while the sampling points of the Yumen and Qiaowan populations were connected through natural river channels. In the cluster analysis, the Yumen and Changma populations clustered into one branch, while the Qiaowan population formed a separate branch. This suggests that the river environment may influence the genetic diversity of *T. tenuis*. In addition, it also indicates that human factors could impact the genetic diversity of natural fish, leading to population differentiation in various environments. Therefore, it is necessary to conduct more extensive and in-depth research on *T. tenuis* to provide reliable scientific guidance for its resource protection and utilization.

## 5 Conclusion

This study analyzed the genetic population structure of the three *T. tenuis* populations from the Shule River Basin, Changma, Yumen, and Qiaowan. It was found that the Changma and Yumen populations shared the origins, while the Qiaowan population was distinct from the former two, which speculated that the populations seemed to diverge in the Shule River Basin. In addition, of the identified 29 polymorphic SSR loci, 15 were able to detect high polymorphisms. The data outputs will contribute to the research on the germplasm resources of *T. tenuis* and the protection of fishery resources in the Shule River.

## Data Availability

The original contributions presented in the study are included in the article/Supplementary Material; further inquiries can be directed to the corresponding authors.
