# Supplementary material for: Analysis of genetic diversity among three Triplophysa tenuis populations by RAD-seq
Source: Front Mol Biosci. 2024 Jul 25;11:1373754. doi: 10.3389/fmolb.2024.1373754 (PMC11308663; doi:10.3389/fmolb.2024.1373754)
Supplement: Supplementary file 1 [file DataSheet1.docx]

Supplemental Material


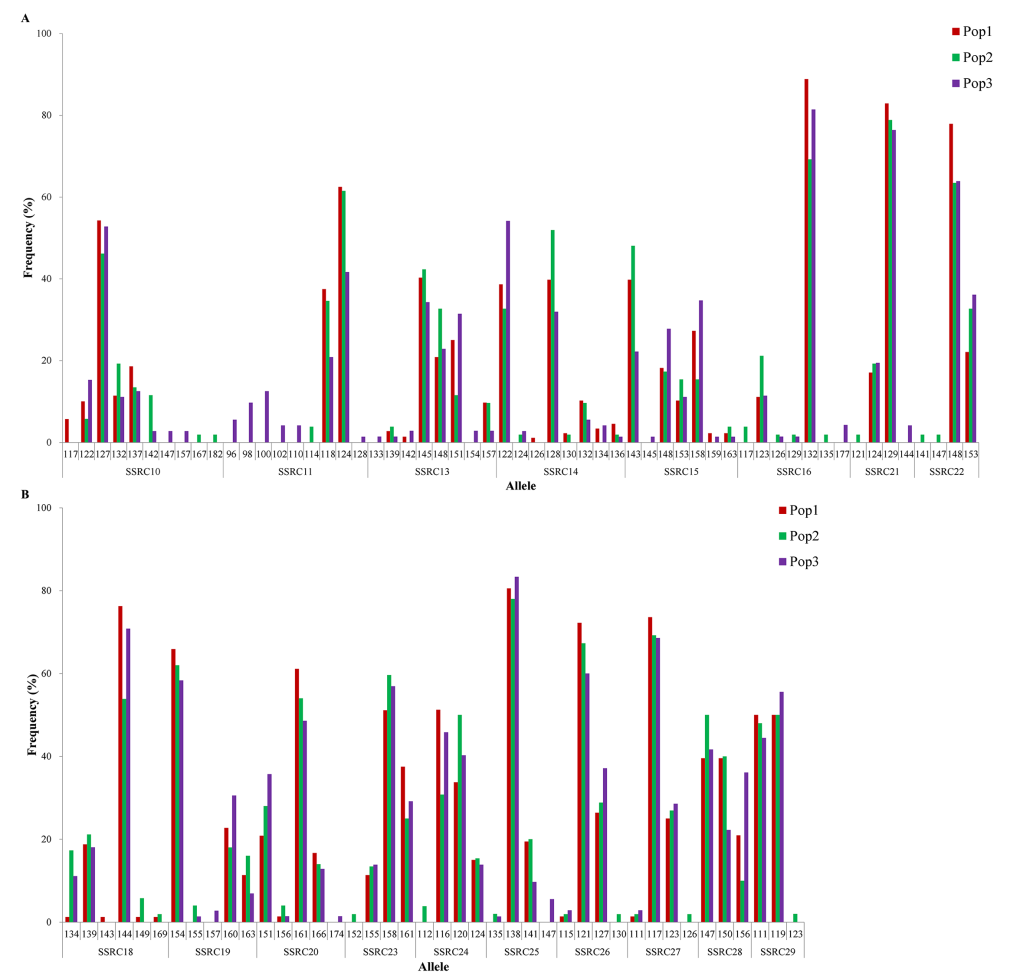


Fig. S1 The allele frequency of SSRs in *T. tenuis*

Note: Pop1, Pop2 and Pop3 represent the population in the Yumen, Changma and Qiaowan, respectively. 122 represents the appearance of SSRC10 at base 122 of the sequence, the same applies to others. The ordinate represents the appearance frequency of SSR.

Table S1 SSRs with polymorphisms and its primers

| Locus | Primer (5’to 3’) f | Primer (5’to 3’) r | Repeating unit | T (℃) | Length (bp) |
| --- | --- | --- | --- | --- | --- |
| C1 | ACCTTGTGCTGAGTGAGGATTTA | CACAAGTGTGAGAATAAACGCAA | （ATCT）7 | 50 | 151~165 |
| C2 | CCAGAAATACTCTGCCATAGTGC | AGGTCAATCTAATCCCTCCTCTG | （AGAT）8 | 53 | 101~157 |
| C3 | ATAAACAGAATGGAGAACTGGCA | TTACACACAAACAGACAGACGGT | （AC）13 | 50 | 115~150 |
| C4 | GGTGTGTAGAGGTGTTTACGGAG | CCCTGAGGGTATTAGAGCTGTTT | （AG）14 | 54 | 139~250 |
| C5 | ATGATAGTTTCTCCTGTGCGTGT | ACATTTGGGTTTCGGTTTAACA | （AT）14 | 49 | 134~171 |
| C6 | TACTCATGCTCCCTCTCTTTGAG | AGCAGCCACTAAATGTAAACGAA | （AG）15 | 51 | 131~162 |
| C7 | GGCCTAAAGGTCAATGCTATTTT | TCCCTTAGTCACACACTCACT | （TGAG ）7 | 50.3 | 117~221 |
| C8 | CGAATATGGGACAGAGAAGAAGA | TGCGCCTGTAAAAACCTACTATT | （AC）13 | 50 | 148~263 |
| C9 | TATAATTGTTCCAGTCTGGCTGC | ATAGCCTGCTGTCCTTTCTCTCT | （AG）13 | 52 | 112~182 |
| C10 | AATTAATTTAGCATTGGCTGGAC | TACTGGATCTAATGGAGCGTGT | （TTGTG）6 | 48.8 | 128~232 |
| C11 | CTCTGATTACCGCAGTGTTTTG | AATCGTGTTTAAGGCAGAAGTCA | （AG）14 | 51 | 112~230 |
| C12 | TTTGGTGTTAATTTTTGCCTACC | CAACTTGACATGGTACAGCTCA | （GTT）7 | 49 | 126~175 |
| C13 | TCCCTGTAAATCTGCTTTGAAAC | CCAGACCTCATAGTTCAGGTAA | （AAT）7 | 50 | 125~149 |
| C14 | TTCCATCTTTTCATATCCCACAC | TGAAAATGAGCTGAATATGTGGA | （AC）12 | 49 | 134~254 |
| C15 | TCAAACCTCCACCATCAAGTATT | TGTGAGGTTGTGTGAAAAGTGTC | （TAAAA）5 | 48.8 | 153~185 |
| C16 | TATTTTGAGGTGGTAAGACAGCG | GCAACCTGCAACATTAACACAAT | （TAT）7 | 50 | 108~128 |
| C17 | AGCTTTGGAGAACAGACGAAAG | CCCATTCTTCATATCCTTTCTGA | （AATG）6 | 49.4 | 150~287 |
| C18 | ATTTTTAAAGTGTCTGCGACGTG | CACTTGCTAATTGCACTTCTGGT | （TAGAT）5 | 48.8 | 146~198 |
| C19 | TCCCACAGACACTACACACAATC | CCAAAACGAAGTACTCTCCACAC | (TCT）9 | 53 | 157~245 |
| C20 | TATTTAATGCCCTTGTGTCTGCT | CAAACACGGTTTAGGCAAGG | （CTTGC）5 | 50 | 60~291 |
| C21 | CACTTGCTAATTGCACTTCTGGT | TCTGTAGCAGTGGAAAGCACAT | （AATCT）5 | 49.4 | 117~132 |
| C22 | CAAAACAATGTAGCATCATGGAA | GAAGAAACAAGCTCAAGGAATCA | （ATTAT）5 | 48.5 | 124~149 |
| C23 | TGTCATTTACATCTTCACAAAGCA | CACAGCACATATCTATGGAGGAA | （TCT）7 | 48.5 | 159~189 |
| C24 | TTTTACAAAAGGTTCTTTGTGGC | TCAAAGGCTCCTTAAGGTTAGGT | （AAGA）6 | 49 | 122~125 |
| C25 | CCTTTCGTGAGTTTACCAAACC | CTTTCTTTAGTGGGTTTATCGGC | （AGA）7 | 51 | 119~141 |
| C26 | GTTAGGGTTCGAGTGAATGTGAA | TCCAGAGTGTCTGAAGACTGATG | （GAT）7 | 52 | 121~246 |
| C27 | TTGAGTTCGAGTGAATGTGAAGA | TCCAGAGTGTCTGAAGACTGAT | （GAT）7 | 50 | 119~291 |
| C28 | AGGATGCAACATGAGAGGAAATA | AATTCATGTTGGAGCATTTATGG | （ATA）7 | 49 | 152~257 |
| C29 | TGATAAACAAGGCCTAAAGGTCA | TCATTCACTCACTACCTTCCTC | （TGAG）7 | 50 | 143~245 |

Table S2 Genetic diversity of the three populations

| Pop | Locus | *N* | *Ne* | *Na* | *Ho* | *He* | *PIC* | *P_HWE_* |
| --- | --- | --- | --- | --- | --- | --- | --- | --- |
| Pop1  (Yumen) | SSRC1 | 36 | 16.615 | 24 | 0.917 | 0.94 | 0.937 | ns |
|  | SSRC2 | 44 | 16 | 21 | 1 | 0.938 | 0.934 | ns |
|  | SSRC4 | 41 | 7.048 | 14 | 0.756 | 0.858 | 0.844 | ns |
|  | SSRC5 | 43 | 6.465 | 13 | 0.86 | 0.845 | 0.829 | ns |
|  | SSRC7 | 43 | 5.519 | 10 | 0.953 | 0.819 | 0.795 | ns |
|  | SSRC8 | 38 | 2.79 | 4 | 0.711 | 0.642 | 0.588 | ns |
|  | SSRC9 | 40 | 2.346 | 6 | 0.65 | 0.574 | 0.517 | ns |
|  | SSRC10 | 35 | 2.813 | 5 | 0.543 | 0.644 | 0.606 | ns |
|  | SSRC11 | 36 | 1.882 | 2 | 0.528 | 0.469 | 0.359 | ns |
|  | SSRC12 | 41 | 2.896 | 4 | 0.78 | 0.655 | 0.594 | ns |
|  | SSRC13 | 36 | 3.59 | 6 | 1 | 0.721 | 0.676 | ns |
|  | SSRC15 | 44 | 3.609 | 6 | 0.977 | 0.723 | 0.678 | ns |
|  | SSRC16 | 36 | 1.246 | 2 | 0.222 | 0.198 | 0.178 | ns |
|  | SSRC17 | 44 | 1.673 | 3 | 0.455 | 0.402 | 0.362 | ns |
|  | SSRC18 | 40 | 1.62 | 6 | 0.475 | 0.383 | 0.341 | ns |
|  | SSRC20 | 36 | 2.248 | 4 | 0.583 | 0.555 | 0.499 | ns |
|  | SSRC21 | 41 | 1.395 | 2 | 0.293 | 0.283 | 0.243 | ns |
|  | SSRC22 | 43 | 1.525 | 2 | 0.395 | 0.344 | 0.285 | ns |
|  | SSRC23 | 44 | 2.409 | 3 | 0.545 | 0.585 | 0.501 | ns |
|  | SSRC24 | 40 | 2.506 | 3 | 0.575 | 0.601 | 0.524 | ns |
|  | SSRC25 | 36 | 1.456 | 2 | 0.333 | 0.313 | 0.264 | ns |
|  | SSRC26 | 36 | 1.691 | 3 | 0.5 | 0.409 | 0.336 | ns |
|  | SSRC27 | 36 | 1.654 | 3 | 0.472 | 0.395 | 0.327 | ns |
|  | SSRC28 | 43 | 2.806 | 3 | 0.651 | 0.644 | 0.567 | ns |
| Average | | 40 | 3.998 | 6.483 | 0.652 | 0.598 | 0.549 |  |
| Pop2  (Changma) | SSRC1 | 26 | 9.013 | 17 | 0.962 | 0.889 | 0.88 | ns |
|  | SSRC2 | 24 | 13.88 | 19 | 0.875 | 0.928 | 0.923 | ns |
|  | SSRC3 | 25 | 6.281 | 12 | 0.88 | 0.841 | 0.824 | ns |
|  | SSRC4 | 25 | 6.545 | 11 | 0.6 | 0.847 | 0.408 | ns |
|  | SSRC6 | 26 | 7.348 | 12 | 0.808 | 0.864 | 0.85 | ns |
|  | SSRC7 | 26 | 4.361 | 8 | 1 | 0.771 | 0.739 | ns |
|  | SSRC8 | 26 | 2.551 | 6 | 0.538 | 0.608 | 0.567 | ns |
|  | SSRC9 | 26 | 3.282 | 6 | 0.577 | 0.695 | 0.648 | ns |
|  | SSRC10 | 26 | 3.503 | 7 | 0.692 | 0.714 | 0.68 | ns |
|  | SSRC12 | 26 | 3.725 | 4 | 0.577 | 0.732 | 0.683 | ns |
|  | SSRC14 | 26 | 2.585 | 6 | 0.577 | 0.613 | 0.548 | ns |
|  | SSRC15 | 26 | 3.227 | 5 | 0.923 | 0.69 | 0.649 | ns |
|  | SSRC20 | 25 | 2.556 | 4 | 0.6 | 0.609 | 0.547 | ns |
|  | SSRC21 | 26 | 1.517 | 3 | 0.346 | 0.341 | 0.295 | ns |
|  | SSRC22 | 26 | 1.959 | 4 | 0.577 | 0.49 | 0.403 | ns |
|  | SSRC23 | 26 | 2.292 | 4 | 0.538 | 0.564 | 0.504 | ns |
|  | SSRC23 | 26 | 3.227 | 5 | 1 | 0.69 | 0.638 | ns |
|  | SSRC25 | 25 | 1.541 | 3 | 0.32 | 0.351 | 0.302 | ns |
|  | SSRC26 | 26 | 1.862 | 4 | 0.5 | 0.463 | 0.387 | ns |
|  | SSRC27 | 26 | 1.81 | 4 | 0.462 | 0.447 | 0.377 | ns |
|  | SSRC28 | 25 | 2.381 | 3 | 0.64 | 0.58 | 0.492 | ns |
| Average | | 26 | 3.691 | 6.414 | 0.645 | 0.638 | 0.574 |  |
| Pop3  (Qiaowan) | SSRC1 | 35 | 13.172 | 21 | 0.943 | 0.924 | 0.919 | ns |
|  | SSRC2 | 35 | 16.225 | 22 | 0.943 | 0.938 | 0.935 | ns |
|  | SSRC3 | 36 | 8.1 | 14 | 0.889 | 0.877 | 0.865 | ns |
|  | SSRC4 | 36 | 8.025 | 14 | 0.861 | 0.875 | 0.863 | ns |
|  | SSRC6 | 35 | 5.724 | 11 | 0.943 | 0.825 | 0.805 | ns |
|  | SSRC5 | 35 | 5.847 | 9 | 0.829 | 0.829 | 0.808 | ns |
|  | SSRC7 | 36 | 4.872 | 10 | 0.639 | 0.795 | 0.772 | ns |
|  | SSRC10 | 36 | 3.01 | 7 | 0.639 | 0.668 | 0.636 | ns |
|  | SSRC13 | 35 | 3.684 | 8 | 1 | 0.729 | 0.681 | ns |
|  | SSRC14 | 36 | 2.492 | 6 | 0.667 | 0.599 | 0.534 | ns |
|  | SSRC15 | 36 | 3.846 | 7 | 1 | 0.74 | 0.695 | ns |
|  | SSRC16 | 35 | 1.474 | 5 | 0.286 | 0.322 | 0.301 | ns |
|  | SSRC18 | 36 | 1.829 | 3 | 0.472 | 0.453 | 0.407 | ns |
|  | SSRC19 | 36 | 2.276 | 5 | 0.583 | 0.561 | 0.492 | ns |
|  | SSRC20 | 35 | 2.629 | 5 | 0.657 | 0.62 | 0.547 | ns |
|  | SSRC21 | 36 | 1.605 | 3 | 0.306 | 0.377 | 0.331 | ns |
|  | SSRC22 | 36 | 1.857 | 2 | 0.5 | 0.461 | 0.355 | ns |
|  | SSRC23 | 36 | 2.333 | 3 | 0.528 | 0.571 | 0.5 | ns |
|  | SSRC24 | 36 | 2.554 | 3 | 0.75 | 0.608 | 0.526 | ns |
|  | SSRC25 | 36 | 1.414 | 4 | 0.333 | 0.293 | 0.275 | ns |
|  | SSRC26 | 35 | 2.005 | 3 | 0.657 | 0.501 | 0.401 | ns |
|  | SSRC27 | 35 | 1.809 | 3 | 0.457 | 0.447 | 0.37 | ns |
|  | SSRC28 | 36 | 2.83 | 3 | 0.722 | 0.647 | 0.571 | ns |
| Average | | 35 | 4.213 | 7.379 | 0.657 | 0.651 | 0.605 |  |

Note: N represents the total number of samples. *Ne* represents the number of effective alleles. *Na* represents the number of alleles. *Ho* represents the observed heterozygosity. *He* represents the expected heterozygosity. PIC represents the polymorphism information content. *P_HWE_* represents the Hardy-Weinberg equilibrium test (with bonferroni correction). All ns values (*p* > 0.05) indicate a locus with genotypes in accordance to Hardy-Weinberg expected proportions (the null hypothesis for the test is HWE).

Table S3 Linkage Disequilibrium analysis of SSR locus in the Yumen population

| Locus | S2 | S19 | S21 | S7 | S17 | S15 | S18 | S22 | S23 | S10 | S3 | S28 | S6 | S14 | S24 | S9 | S11 | S4 | S12 | S13 | S20 | S25 | S29 | S26 | S1 | S16 | S27 | S8 | S5 |
| --- | --- | --- | --- | --- | --- | --- | --- | --- | --- | --- | --- | --- | --- | --- | --- | --- | --- | --- | --- | --- | --- | --- | --- | --- | --- | --- | --- | --- | --- |
| S2 | * | - | - | - | - | - | - | - | - | - | - | - | - | - | - | - | - | - | - | - | - | - | - | - | - | - | - | - | - |
| S19 |  | * | - | - | - | - | - | - | - | - | - | - | - | - | - | + | - | + | - | - | - | - | - | - | - | - | - | - | - |
| S21 |  |  | * | - | - | - | + | - | - | - | + | - | - | - | - | - | - | - | - | - | - | - | - | - | - | - | - | - | - |
| S7 |  |  |  | * | - | - | - | - | - | - | - | - | - | - | - | - | - | - | - | - | - | - | - | - | - | - | - | - | - |
| S17 |  |  |  |  | * | - | - | - | - | - | - | - | - | - | - | - | - | - | - | - | - | - | + | - | - | - | - | - | + |
| S15 |  |  |  |  |  | * | - | - | - | - | - | - | - | - | + | - | - | - | - | - | - | - | + | - | - | - | - | - | - |
| S18 |  |  |  |  |  |  | * | - | - | - | - | - | - | - | - | - | - | - | - | - | - | - | - | - | - | - | - | - | - |
| S22 |  |  |  |  |  |  |  | * | - | - | - | - | - | - | - | - | - | - | - | - | - | - | - | - | - | - | - | - | - |
| S23 |  |  |  |  |  |  |  |  | * | - | - | - | - | - | - | - | - | - | - | - | - | - | - | - | - | - | - | - | - |
| S10 |  |  |  |  |  |  |  |  |  | * | - | - | - | - | + | + | + | + | + | + | + | + | + | + | + | + | + | + | - |
| S3 |  |  |  |  |  |  |  |  |  |  | * | - | - | - | + | - | + | - | - | - | + | + | + | - | - | + | - | + | - |
| S28 |  |  |  |  |  |  |  |  |  |  |  | * | - | + | + | - | - | - | - | - | - | - | - | - | - | + | - | + | - |
| S6 |  |  |  |  |  |  |  |  |  |  |  |  | * | - | - | - | - | - | - | - | - | - | - | - | - | - | - | - | + |
| S14 |  |  |  |  |  |  |  |  |  |  |  |  |  | * | + | - | - | - | + | - | - | - | - | - | - | - | - | - | - |
| S24 |  |  |  |  |  |  |  |  |  |  |  |  |  |  | * | + | + | + | + | + | + | + | + | + | + | + | + | + | - |
| S9 |  |  |  |  |  |  |  |  |  |  |  |  |  |  |  | * | + | + | + | + | + | + | + | + | + | + | + | + | + |
| S11 |  |  |  |  |  |  |  |  |  |  |  |  |  |  |  |  | * | + | + | + | + | + | + | + | + | + | + | + | - |
| S4 |  |  |  |  |  |  |  |  |  |  |  |  |  |  |  |  |  | * | + | + | + | + | + | + | + | + | + | + | - |
| S12 |  |  |  |  |  |  |  |  |  |  |  |  |  |  |  |  |  |  | * | + | + | + | + | + | + | + | + | + | - |
| S13 |  |  |  |  |  |  |  |  |  |  |  |  |  |  |  |  |  |  |  | * | + | + | + | + | + | + | + | + | - |
| S20 |  |  |  |  |  |  |  |  |  |  |  |  |  |  |  |  |  |  |  |  | * | + | + | + | + | + | + | + | - |
| S25 |  |  |  |  |  |  |  |  |  |  |  |  |  |  |  |  |  |  |  |  |  | * | + | + | + | + | + | + | + |
| S29 |  |  |  |  |  |  |  |  |  |  |  |  |  |  |  |  |  |  |  |  |  |  | * | + | + | + | + | + | - |
| S26 |  |  |  |  |  |  |  |  |  |  |  |  |  |  |  |  |  |  |  |  |  |  |  | * | + | + | + | + | + |
| S1 |  |  |  |  |  |  |  |  |  |  |  |  |  |  |  |  |  |  |  |  |  |  |  |  | * | + | + | + | - |
| S16 |  |  |  |  |  |  |  |  |  |  |  |  |  |  |  |  |  |  |  |  |  |  |  |  |  | * | + | + | - |
| S27 |  |  |  |  |  |  |  |  |  |  |  |  |  |  |  |  |  |  |  |  |  |  |  |  |  |  | * | + | + |
| S8 |  |  |  |  |  |  |  |  |  |  |  |  |  |  |  |  |  |  |  |  |  |  |  |  |  |  |  | * | - |
| S5 |  |  |  |  |  |  |  |  |  |  |  |  |  |  |  |  |  |  |  |  |  |  |  |  |  |  |  |  | * |

Note: “S2, S19, S21, S7, S17, S15, S18, S22, S23, S10, S3, S28, S6, S14, S24, S9, S11, S4, S12, S13, S20, S25, S29, S26, S1, S16, S27, S8, and S5” represent “SSRC2, SSRC19, SSRC21, SSRC7, SSRC17, SSRC15, SSRC18, SSRC22, SSRC23, SSRC10, SSRC3, SSRC28, SSRC6, SSRC14, SSRC24, SSRC9, SSRC11, SSRC4, SSRC12, SSRC13, SSRC20, SSRC25, SSRC29, SSRC26, SSRC1, SSRC16, SSRC27, SSRC8 and SSRC5, respectively. “+” and “-” represent two SSR loci linkage unbalance and linkage balance, respectively.

Table S4 Linkage Disequilibrium analysis of SSR locus in the Cahngma population

| Locus | S2 | S19 | S21 | S7 | S17 | S15 | S18 | S22 | S23 | S10 | S3 | S28 | S6 | S14 | S24 | S9 | S11 | S4 | S12 | S13 | S20 | S25 | S29 | S26 | S1 | S16 | S27 | S8 | S5 |
| --- | --- | --- | --- | --- | --- | --- | --- | --- | --- | --- | --- | --- | --- | --- | --- | --- | --- | --- | --- | --- | --- | --- | --- | --- | --- | --- | --- | --- | --- |
| S2 | * | - | - | - | - | - | - | - | - | - | - | - | - | - | - | - | - | - | - | - | - | - | - | - | + | - | - | - | - |
| S19 |  | * | - | - | - | - | - | - | - | - | - | + | - | + | - | - | - | + | - | - | - | + | - | - | + | + | - | - | - |
| S21 |  |  | * | - | - | - | - | - | - | - | - | - | - | - | - | - | - | - | - | - | - | + | - | - | - | - | - | - | - |
| S7 |  |  |  | * | - | - | - | - | - | - | - | - | - | - | - | - | - | - | - | - | - | - | + | - | - | - | + | - | - |
| S17 |  |  |  |  | * | - | - | - | - | - | + | - | - | - | - | - | + | - | - | - | - | - | - | - | - | - | - | - | - |
| S15 |  |  |  |  |  | * | - | - | + | - | - | - | - | - | - | - | - | - | - | - | - | - | + | + | - | - | + | - | - |
| S18 |  |  |  |  |  |  | * | + | - | - | - | - | - | - | - | - | - | - | - | - | - | - | - | - | - | - | - | - | - |
| S22 |  |  |  |  |  |  |  | * | - | - | - | - | - | - | - | - | - | - | - | - | - | - | - | - | - | - | - | - | - |
| S23 |  |  |  |  |  |  |  |  | * | - | - | - | - | - | - | - | - | - | + | - | - | - | - | - | - | - | - | - | - |
| S10 |  |  |  |  |  |  |  |  |  | * | - | - | - | - | - | - | - | - | - | - | - | - | - | - | - | - | - | - | - |
| S3 |  |  |  |  |  |  |  |  |  |  | * | + | - | - | - | - | - | - | - | - | - | + | - | - | - | + | - | - | - |
| S28 |  |  |  |  |  |  |  |  |  |  |  | * | - | - | - | - | - | + | - | - | - | - | - | - | - | + | - | - | + |
| S6 |  |  |  |  |  |  |  |  |  |  |  |  | * | - | + | - | + | - | - | - | - | + | - | - | - | + | - | - | - |
| S14 |  |  |  |  |  |  |  |  |  |  |  |  |  | * | - | - | + | - | - | - | + | + | + | - | - | + | - | - | - |
| S24 |  |  |  |  |  |  |  |  |  |  |  |  |  |  | * | - | + | - | - | - | + | + | + | - | + | + | - | - | - |
| S9 |  |  |  |  |  |  |  |  |  |  |  |  |  |  |  | * | + | - | - | - | - | - | + | - | - | + | - | - | - |
| S11 |  |  |  |  |  |  |  |  |  |  |  |  |  |  |  |  | * | - | - | - | + | + | + | - | - | + | - | - | + |
| S4 |  |  |  |  |  |  |  |  |  |  |  |  |  |  |  |  |  | * | - | - | - | - | - | - | - | + | - | - | - |
| S12 |  |  |  |  |  |  |  |  |  |  |  |  |  |  |  |  |  |  | * | - | - | + | - | - | + | - | - | - | - |
| S13 |  |  |  |  |  |  |  |  |  |  |  |  |  |  |  |  |  |  |  | * | - | - | + | - | - | - | - | + | - |
| S20 |  |  |  |  |  |  |  |  |  |  |  |  |  |  |  |  |  |  |  |  | * | + | + | - | - | + | - | - | - |
| S25 |  |  |  |  |  |  |  |  |  |  |  |  |  |  |  |  |  |  |  |  |  | * | + | - | - | + | - | - | + |
| S29 |  |  |  |  |  |  |  |  |  |  |  |  |  |  |  |  |  |  |  |  |  |  | * | - | - | + | - | - | + |
| S26 |  |  |  |  |  |  |  |  |  |  |  |  |  |  |  |  |  |  |  |  |  |  |  | * | - | - | + | - | - |
| S1 |  |  |  |  |  |  |  |  |  |  |  |  |  |  |  |  |  |  |  |  |  |  |  |  | * | - | - | - | - |
| S16 |  |  |  |  |  |  |  |  |  |  |  |  |  |  |  |  |  |  |  |  |  |  |  |  |  | * | - | - | - |
| S27 |  |  |  |  |  |  |  |  |  |  |  |  |  |  |  |  |  |  |  |  |  |  |  |  |  |  | * | - | - |
| S8 |  |  |  |  |  |  |  |  |  |  |  |  |  |  |  |  |  |  |  |  |  |  |  |  |  |  |  | * | - |
| S5 |  |  |  |  |  |  |  |  |  |  |  |  |  |  |  |  |  |  |  |  |  |  |  |  |  |  |  |  | * |

Note: “S2, S19, S21, S7, S17, S15, S18, S22, S23, S10, S3, S28, S6, S14, S24, S9, S11, S4, S12, S13, S20, S25, S29, S26, S1, S16, S27, S8, and S5” represent “SSRC2, SSRC19, SSRC21, SSRC7, SSRC17, SSRC15, SSRC18, SSRC22, SSRC23, SSRC10, SSRC3, SSRC28, SSRC6, SSRC14, SSRC24, SSRC9, SSRC11, SSRC4, SSRC12, SSRC13, SSRC20, SSRC25, SSRC29, SSRC26, SSRC1, SSRC16, SSRC27, SSRC8 and SSRC5, respectively. “+” and “-” represent two SSR loci linkage unbalance and linkage balance, respectively.

Table S5 Linkage Disequilibrium analysis of SSR locus in the Qiaowan population

| Locus | S2 | S19 | S21 | S7 | S17 | S15 | S18 | S22 | S23 | S10 | S3 | S28 | S6 | S14 | S24 | S9 | S11 | S4 | S12 | S13 | S20 | S25 | S29 | S26 | S1 | S16 | S27 | S8 | S5 |
| --- | --- | --- | --- | --- | --- | --- | --- | --- | --- | --- | --- | --- | --- | --- | --- | --- | --- | --- | --- | --- | --- | --- | --- | --- | --- | --- | --- | --- | --- |
| S2 | * | - | - | - | - | - | + | - | - | - | - | - | - | - | - | - | - | + | - | - | - | - | - | - | - | - | - | - | - |
| S19 |  | * | - | - | - | - | - | - | - | - | - | - | - | - | - | - | - | - | - | - | - | - | - | - | - | - | - | - | - |
| S21 |  |  | * | - | - | - | + | - | - | - | - | - | - | - | + | - | + | - | - | - | - | - | - | - | - | - | - | + | - |
| S7 |  |  |  | * | - | - | - | - | - | - | - | - | - | - | - | + | + | - | + | + | - | - | - | - | - | - | - | + | - |
| S17 |  |  |  |  | * | - | - | - | - | - | - | - | - | - | - | - | - | - | - | - | - | - | - | - | - | - | - | - | - |
| S15 |  |  |  |  |  | * | - | - | - | - | - | - | - | - | - | - | - | - | - | - | - | - | + | - | - | - | - | - | - |
| S18 |  |  |  |  |  |  | * | - | - | - | - | - | - | - | - | - | - | - | - | + | - | - | - | - | - | - | - | - | - |
| S22 |  |  |  |  |  |  |  | * | - | - | - | - | - | - | - | - | - | - | - | - | - | - | - | - | - | - | - | - | - |
| S23 |  |  |  |  |  |  |  |  | * | - | - | - | - | - | - | - | - | - | - | - | - | - | - | - | - | - | - | - | - |
| S10 |  |  |  |  |  |  |  |  |  | * | - | - | - | - | - | - | - | - | + | - | - | - | - | - | - | - | - | + | - |
| S3 |  |  |  |  |  |  |  |  |  |  | * | - | - | - | - | - | - | - | - | - | - | - | + | - | - | - | - | + | - |
| S28 |  |  |  |  |  |  |  |  |  |  |  | * | - | - | - | - | - | - | - | - | - | - | - | - | - | - | - | - | - |
| S6 |  |  |  |  |  |  |  |  |  |  |  |  | * | - | - | - | - | - | - | + | - | - | + | - | - | - | - | - | - |
| S14 |  |  |  |  |  |  |  |  |  |  |  |  |  | * | - | - | - | - | - | - | - | - | - | - | - | - | - | - | + |
| S24 |  |  |  |  |  |  |  |  |  |  |  |  |  |  | * | - | + | - | + | - | - | - | - | + | - | - | - | - | - |
| S9 |  |  |  |  |  |  |  |  |  |  |  |  |  |  |  | * | + | - | + | - | - | - | - | - | - | - | - | - | + |
| S11 |  |  |  |  |  |  |  |  |  |  |  |  |  |  |  |  | * | - | + | - | - | - | - | - | - | - | - | - | + |
| S4 |  |  |  |  |  |  |  |  |  |  |  |  |  |  |  |  |  | * | - | - | - | - | - | - | - | - | - | - | - |
| S12 |  |  |  |  |  |  |  |  |  |  |  |  |  |  |  |  |  |  | * | - | + | + | - | + | - | + | - | + | - |
| S13 |  |  |  |  |  |  |  |  |  |  |  |  |  |  |  |  |  |  |  | * | - | - | + | - | - | - | - | - | - |
| S20 |  |  |  |  |  |  |  |  |  |  |  |  |  |  |  |  |  |  |  |  | * | - | - | + | + | + | + | - | - |
| S25 |  |  |  |  |  |  |  |  |  |  |  |  |  |  |  |  |  |  |  |  |  | * | - | - | - | - | - | + | - |
| S29 |  |  |  |  |  |  |  |  |  |  |  |  |  |  |  |  |  |  |  |  |  |  | * | - | - | - | - | + | - |
| S26 |  |  |  |  |  |  |  |  |  |  |  |  |  |  |  |  |  |  |  |  |  |  |  | * | - | + | + | - | - |
| S1 |  |  |  |  |  |  |  |  |  |  |  |  |  |  |  |  |  |  |  |  |  |  |  |  | * | - | - | - | + |
| S16 |  |  |  |  |  |  |  |  |  |  |  |  |  |  |  |  |  |  |  |  |  |  |  |  |  | * | + | + | - |
| S27 |  |  |  |  |  |  |  |  |  |  |  |  |  |  |  |  |  |  |  |  |  |  |  |  |  |  | * | - | - |
| S8 |  |  |  |  |  |  |  |  |  |  |  |  |  |  |  |  |  |  |  |  |  |  |  |  |  |  |  | * | + |
| S5 |  |  |  |  |  |  |  |  |  |  |  |  |  |  |  |  |  |  |  |  |  |  |  |  |  |  |  |  | * |

Note: “S2, S19, S21, S7, S17, S15, S18, S22, S23, S10, S3, S28, S6, S14, S24, S9, S11, S4, S12, S13, S20, S25, S29, S26, S1, S16, S27, S8, and S5” represent “SSRC2, SSRC19, SSRC21, SSRC7, SSRC17, SSRC15, SSRC18, SSRC22, SSRC23, SSRC10, SSRC3, SSRC28, SSRC6, SSRC14, SSRC24, SSRC9, SSRC11, SSRC4, SSRC12, SSRC13, SSRC20, SSRC25, SSRC29, SSRC26, SSRC1, SSRC16, SSRC27, SSRC8 and SSRC5, respectively. “+” and “-” represent two SSR loci linkage unbalance and linkage balance, respectively.
